# Exploring the Feasibility of P7 Medicine Principles in Dental Practice (P7 Dentistry) 

**DOI:** 10.30476/dentjods.2025.108949.2858

**Published:** 2026-03-01

**Authors:** Milad Saeedzadeh, Shahram Hamedani, Saeed Nemati

**Affiliations:** 1 Student Research Committee, Shiraz University of Medical Sciences, Shiraz, Iran.; 2 Oral and Dental Disease Research Center, School of Dentistry, Shiraz University of Medical Sciences, Shiraz, Iran.; 3 Shiraz University of Medical Sciences, Shiraz, Iran.

**Dear Editor**

Through two decades ago, the concept of health care was close to reactive medicine and its focus was on treatment rather than proactive activities. In other words, instead of an individual-centric system, it was a disease-centric system [ 1 ]. This system gradually changed with advances in technology and biology. The data gained from these developments led to the emergence of an approach that was “Predictive”, “Personalized”, “Preventive”, and “Participatory”, which was referred as “P4 medicine”. This approach through analyzing the data could detect and treat disorder long before disease symptoms appear and reduce the cost of health care. It also enabled a team of specialists to collaborate in the treatment by sharing data, rather than leaving the responsibility to a single doctor. Hence, a wellness model has emerged whereby the objective of patient management was to prioritize comprehensive well-being of the patient rather than merely treat a disease and its symptoms [ 2 ].

Despite the benefits of P4 medicine, it has faced criticism, mainly for reducing patients to biological data and ignoring their personal values, desires, and beliefs, raising concerns about depersonalization. To overcome these shortcomings, three additional components including population, psycho-cognitive, and public health have been added to P4 medicine to create a more complete concept, currently known as “P7 medicine”. This system represents a framework which individualizes the healthcare with respect to its biological, psychological, and social components. Consequently, it presents the individual not simply as the center of the clinical encounter, but as one element within the healthcare system [ 3 ].

By expanding the P4 medicine concept and integrating it with the field of oral health, a personalized, predictive, preventive, and participatory system was also created in dentistry which was named as “P4 dentistry” [ 1 ]. This system reduces the often destructive and chronic nature of these disorders by prioritizing a preventive approach to diagnosis and treatment, rather than the current reactive, wait-and-see (monitoring) approach. P4 dentistry provides individuals with customized treatments and supportive cares intervals designed to prevent dental problems. It also improves treatment outcomes and can effectively educate patients about their health. Furthermore, the expansion of technological capabilities, coupled with the rise of teledentistry has made it feasible to remotely collect and document a broad range of clinical, imaging, and laboratory data from patients [ 4 ]. This makes it possible to transfer the customized diagnostic, preventive, and therapeutic pathways from the hospital setting to the home. Such data can be easily shared among specialists and would enhance the comprehensive management of complex cases within multidisciplinary teams [ 1 ]. But this system still has some drawbacks as it treats individuals as a “collection of data”. By adding population, public health, and psychology to this concept and upgrading it to P7 dentistry, these disadvantages can be overcome, as was previously done for P4 medicine. With these criticisms resolved, one of the problems that could stand in the way of P7 dentistry is the feasibility of this concept in a clinical setting, which requires analyzing large amounts of data and reaching the best decisions for the patient. One of the most effective tools that can solve this problem is the use of artificial intelligence (AI), which can empower personalized dental workflows by analyzing comprehensive health data of an individual patient and determine optimized treatment plans and risk management on a case-by-case basis. For instance, emerging AI systems are expected to calculate the specific risk of tooth loss regarding preventive factors such as cleaning ability, caries activity, and access to dental care [ 5 ]. 

Predictions of tooth prognosis can be made by considering each patient’s dental condition along with broader social and medical factors, using insights from big data to guide clinicians in personalized prevention and treatment planning. AI can also detect hidden patterns of concurrent diseases that may affect clinical outcomes, which clinicians might not easily recognize. Therefore, it has great potential to assist dental practitioners in treatment planning, reduce time-consuming tasks, and identify details that could otherwise be overlooked [ 5 ].

We encourage dedicated research on P7 dentistry to be pursued to achieve comprehensive, patient-centered, and population-oriented oral care. Meanwhile, the use of AI can provide more accurate and complete results by analyzing data. First, the integration of AI into clinical practice can streamline complex decision-making, allowing clinicians to focus on personalized care rather than administrative tasks. Second, ongoing training and collaboration among multidisciplinary teams are essential to maximize the potential of P7 dentistry. Third, ethical considerations and patient consent must remain central when using AI and big data to ensure care remains truly patient-centered.
